# Factors Influencing Postoperative Complications Following Minimally Invasive Ivor Lewis Esophagectomy: A Retrospective Cohort Study

**DOI:** 10.3390/jcm12175688

**Published:** 2023-08-31

**Authors:** Antje K. Peters, Mazen A. Juratli, Dhruvajyoti Roy, Jennifer Merten, Lukas Fortmann, Andreas Pascher, Jens Peter Hoelzen

**Affiliations:** 1 Department of General, Visceral and Transplant Surgery, University Hospital Muenster, 48149 Muenster, Germany; antje.peters@uni-muenster.de (A.K.P.); mazen.juratli@ukmuenster.de (M.A.J.);; 2 Institute of Medical Psychology and Systems Neuroscience, University of Muenster, 48149 Muenster, Germany; 3 Otto Creutzfeldt Center for Cognitive and Behavioral Neuroscience, University of Muenster, 48149 Muenster, Germany; 4 Department of Surgical Oncology, University of Texas MD Anderson Cancer Center, Houston, TX 77030, USA

**Keywords:** minimally invasive Ivor Lewis esophagectomy, RAMIE, enteral nutrition, percutaneous endoscopic jejunostomy (PEJ), anastomotic insufficiency

## Abstract

Background: Complications arising following minimally invasive Ivor Lewis esophagectomy often result from inadequate enteral nutrition, highlighting the need for proactive measures to prevent such issues. One approach involves identifying high-risk cases prone to complications and implementing percutaneous endoscopic jejunostomy (PEJ) tube placement during esophageal resection to ensure timely enteral nutrition. Methods: In this single-center, retrospective cohort study, we examined patients who underwent minimally invasive esophagectomy for esophageal cancer at a high-volume center. The dataset encompassed demographic information, comorbidities, laboratory parameters, and intraoperative details. Our center utilized the EndoVac system pre-emptively to safeguard the anastomosis from harmful secretions and to enhance local oxygen partial pressure. All patients received pre-emptive EndoVac therapy and underwent esophagogastroduodenoscopy in the early postoperative days. The need for multiple postoperative EndoVac cycles indicated complications, including anastomotic insufficiency and subsequent requirement for a PEJ. The primary objectives were identifying predictive factors for anastomotic insufficiency and the need for multi-cycle EndoVac therapy, quantifying their effects, and assessing the likelihood of postoperative complications. Results: 149 patients who underwent minimally invasive or hybrid Ivor Lewis esophagectomy were analyzed and 21 perioperative and demographic features were evaluated. Postoperative complications were associated with the body mass index (BMI) category, the use of blood pressure medication, and surgery duration. Anastomotic insufficiency as a specific complication was correlated with BMI and the Charlson comorbidity index. The odds ratio of being in the high-risk group significantly increased with higher BMI (OR = 1.074, *p* = 0.048) and longer surgery duration (OR = 1.005, *p* = 0.004). Conclusions: Based on our findings, high BMI and longer surgery duration are potential risk factors for postoperative complications following minimally invasive esophagectomy. Identifying such factors can aid in pre-emptively addressing nutritional challenges and reducing the incidence of complications in high-risk patients.

## 1. Introduction

Esophageal carcinomas are the sixth leading cause of cancer mortality worldwide [1], and their incidence in the Western population is increasing [2]. Minimally invasive Ivor Lewis esophagectomy is a surgical procedure commonly performed to treat esophageal cancer [3,4,5,6]. While this technique offers numerous benefits, including reduced postoperative pain and shorter hospital stays, post-surgery complications, such as anastomotic insufficiency (AI), remain a significant concern, with an incidence of 11.4–21.2 percent [7]. Among the key challenges is the inability to provide adequate enteral nutrition during recovery, leading to potential complications and delayed healing of anastomotic sites. One approach for mitigating these issues involves identifying cases at high risk of complications and implementing feeding tubes, such as percutaneous endoscopic jejunostomy (PEJ) tubes, during esophageal resection. This strategy ensures timely enteral nutrition delivery, hence reducing the likelihood of postoperative complications.

Our high-volume center has adopted a pioneering approach over the past decade by utilizing a pre-emptive EndoVac sponge, following each esophagectomy with intrathoracic anastomosis. After each esophageal resection, esophagogastroduodenoscopy is performed and a diameter-matched sponge is positioned on the anastomosis under visualization using a retraction technique. After 5 days, the EndoVac sponge is removed and a control endoscopy is performed. In case of irregularities, an individually trimmed sponge is placed again. This proactive measure has proven beneficial in preventing potential complications. In certain cases, extended EndoVac therapy is required, particularly in instances of AI. When postoperative complications necessitate the prolonged use of EndoVac therapy, enteral therapy is recommended as a complementary intervention [8]. As parenteral nutrition carries the potential risks of hyperglycemia, hypertriglyceridemia, electrolyte imbalances, and long-term hepatobiliary and bone diseases [9], alternative nutritional strategies are sought to ensure optimal patient outcomes. Among these, enteral nutrition is preferred [10], and PEJ is mainly considered for patients at high risk of AI [11]. However, the lack of a standardized postoperative PEJ insertion procedure raises concerns about the timing and necessity of this intervention, as the process can be burdensome for patients: On the one hand, inserting a PEJ tube has risks, including potential complications such as aspiration pneumonia, wound infection, and bleeding [12]. These risks argue against an overly generous use of a tube. On the other hand, if a patient develops postoperative complications, a separate surgery is required for PEJ tube insertion, adding to the overall complexity of their care. This consideration would argue for the simultaneous insertion of a probe.

To address this ambiguity, there is a growing interest in predicting the likelihood of post-esophagectomy complications based on perioperative and demographic patient data. By identifying patients at high risk of complications, healthcare professionals can make informed decisions and potentially perform PEJ tube insertion during the esophagectomy, streamlining the process and minimizing the need for additional surgeries. Such predictive tools offer the potential to enhance patient care by optimizing nutritional support and reducing the impact of complications, ultimately leading to improved postoperative outcomes.

This single-center, retrospective cohort study investigated factors influencing postoperative complications such as AI following minimally invasive Ivor Lewis esophagectomy. This study was unique because it was able to draw conclusions from a local population in which pre-emptive EndoVac therapy and regular endoscopy were performed. We hypothesized that the data collected would identify risk factors associated with postoperative complications. The study aimed to identify predictors of AI as a specific and very common complication. However, AI affects only a few cases of the total sample, which makes the evaluation challenging. Therefore, we introduced the need for multiple cycles of EndoVac therapy as a surrogate parameter for a complicative course. This event occurs more often and is more straightforward to address.

## 2. Materials and Methods

Patients: This retrospective cohort study comprised 149 patients who underwent either minimally invasive or hybrid Ivor Lewis esophagectomy for esophageal cancer at Münster University Hospital between February 2012 and March 2022. We included all patients aged 18 years or older with thoracic or abdominal esophagus carcinoma that was both histologically diagnosed and resectable. In the case of neoadjuvant therapy, respectability after therapy was decisive. Cases where surgery could not be completed or where laparoscopic intervention became necessary were excluded. The study also excluded patients with evidence of COVID-19 infection and those undergoing two-stage operations. All included patients had undergone postoperative pre-emptive EndoVac insertion. For this purpose, at the end of the operation, and before removing the double-lumen tube for ventilation, an esophagogastroduodenoscopy was performed and the anastomosis was examined. An EndoVac sponge was then cut to fit the diameter of the gastric tube and positioned at the level of the anastomosis using the retraction technique under visual control. The tube was then fed out of the nose, connected to a suction generator with a suction of −125 mmHg, and fixed to the nose. After 5 days, the sponge was removed and control endoscopy was performed under short-term anesthesia. In case of irregularities in anastomosis healing, for example, a widened anastomosis or visible staples, prophylactic therapy was continued in 5-day cycles. EndoVac therapy was continued in the event of an anastomotic leak, albeit not as a prevention but as a therapy. Where this therapeutical approach was used, esophagogastroduodenoscopy was performed and repeated every 5 days. Diagnostics for cancer staging were performed based on physical and nutritional assessments, endoscopy (including biopsy), endoscopic ultrasound, and computer tomography scan. A multidisciplinary cancer board decided on surgery, and neoadjuvant treatments were administered following the German Cancer Society (DKG) guidelines for esophageal adenocarcinoma and squamous cell carcinoma, using FLOT (fluorouracil/leucovorin/oxaliplatin/docetaxel) or CROSS (carboplatin/paclitaxel) treatment schemes [13,14].

All procedures were conducted using minimally invasive techniques, either as hybrid esophagectomy (laparoscopic gastric mobilization and open right thoracotomy) or robot-assisted minimally invasive esophagectomies (RAMIEs) utilizing the da Vinci Surgery System (Intuitive Surgical Inc., Sunnyvale, CA, USA). As a high-volume center with many years of experience in esophageal surgery, surgical methods were changed to minimally invasive procedures after the publication of the Time Study [15]. In 2018, the robot-assisted technique (RAMIE) was introduced. The MIRO Trial [16], ROBOT Trial [17], and RAMIE Trial [18] showed the advantages of robot-assisted procedures. All procedures had the minimally invasive approach in common. Resection and anastomosis were the same, with different access routes, and, therefore, had comparable risks. In this regard, our sample represents a homogeneous collective. Gastrolysis, gastric tube formation, and D2 lymphadenectomy were performed in all surgical procedures. At the end of the abdominal phase, an initial vascularization check of the gastric tube was performed with ICG. In the thoracic part, an en bloc esophagectomy with lymphadenectomy and appropriate safety margin was performed and checked using frozen sections. Anastomosis of the gastric tube with the remaining esophagus was performed using an end-to-side technique using a 29 mm circular stapler. After completing the anastomosis, another vascularization check was performed with ICG. For more details on the surgical procedures (hybrid-esophagectomy and RAMIE), refer to Ref [4].

Clinical characteristics, surgery details, and preoperative laboratory parameters were extracted from hospital records. Patients were followed up for at least 30 days to monitor postoperative complications. The dataset contained 21 features, as listed in Table 1. All procedures adhered to the Declaration of Helsinki with Good Clinical Practice (GCP) and the STROCSS 2019 Guideline [19,20,21]. Ethical approval was obtained from the combined ethics committee of the University of Muenster (Muenster, Germany) and the Medical Association of Westphalia-Lippe (reference number: 2022-123-f-S), and written general consent for the scientific use of medical data was obtained from all patients.

Endpoints: At Münster University Hospital, a proactive approach is implemented to minimize postoperative complications after esophagectomy. Patients undergo pre-emptive EndoVac therapy, which involves the placement of an EndoVac intraoperatively. This approach contrasts with the standard procedure, where the EndoVac is used only in case of complications [22]. The EndoVac is a standard therapy for postoperative treatment [23,24,25]. The pre-emptive EndoVac therapy is a novel technology for reducing AI rate and postoperative morbidity. The approach has also been used successfully in other centers [26,27,28]. Following surgery, patients are closely monitored in the Intensive Care Unit (ICU) for at least one night, with extended stay if necessary. Once there are no complications, patients are transferred to the general ward, where they receive standardized postoperative care.

All patients undergo esophagogastroduodenoscopy on the fifth day after surgery to check for AI and identify any defects based on the Esophagectomy Complications Consensus Group standards [29]. If the findings are normal, the EndoVac is removed and the patient is started on an oral diet. The occurrence of AI is defined as the first endpoint in this investigation. However, in cases where complications—including but not limited to AI—arise, EndoVac therapy is continued. If the EndoVac probe remains in place beyond the fifth day, enteral nutrition is given using a PEJ tube (Freka FCJ FR 9, Fresenius Kabi, Bad Homburg, Germany). Note that, in principle, a feeding tube can be placed along the sponge of the EndoVac. However, this is disadvantageous because pressure points and concomitant local reduced blood flow delay the healing of the surgical area. In this regard, a PEJ tube is chosen to provide nutrition via enteral feeding, which is the standard after esophagectomy [30,31]. During laparotomic esophagectomy, PEJ tube insertion is recommended for patients regardless of complications [32].

The need for one or more EndoVac changes beyond the single prophylactic EndoVac cycle indicates a complicated course and defines the second endpoint. Based on the number of EndoVac cycles required, patients can be categorized into low-risk or high-risk groups, allowing for further risk stratification. This proactive approach aims to optimize patient outcomes by promptly identifying and managing complications, ultimately enhancing the success of esophagectomy procedures.

Statistical analysis: Statistical analyses were performed in Python 3 to explore the different feature expressions within the two classes defined by the endpoints. Statistical significance was determined using the chi-square test to compare two proportions to test the hypothesis that proportions do not differ between the high and low-risk groups. The test was performed with Scipy using the statsmodels toolbox [33]. We performed Welch’s *t*-test to test two independent samples’ average (expected) values for equality. We chose Welch’s *t*-test as it is less biased compared with the Student’s *t*-test in cases of non-equal variance in the compared datasets [34]. The *t*-test was performed using Python’s Pingouin package [35]. Binomial logistic regression was performed to determine the ability of certain correlated features to predict the dichotomous variable of the investigated cases’ group membership (high or low-risk group). These features are the laboratory and patient data specified in the results section. The logistic regression assigns weights to the respective features. It also assigns the odds of being in the high-risk group based on the features. We used the statsmodels’ Logit function to perform logistic regression. All reported *p*-values are two-sided. The significance level was set to *p* = 0.05.

## 3. Results

### 3.1. Preoperative Characteristics

**Figure 1:**
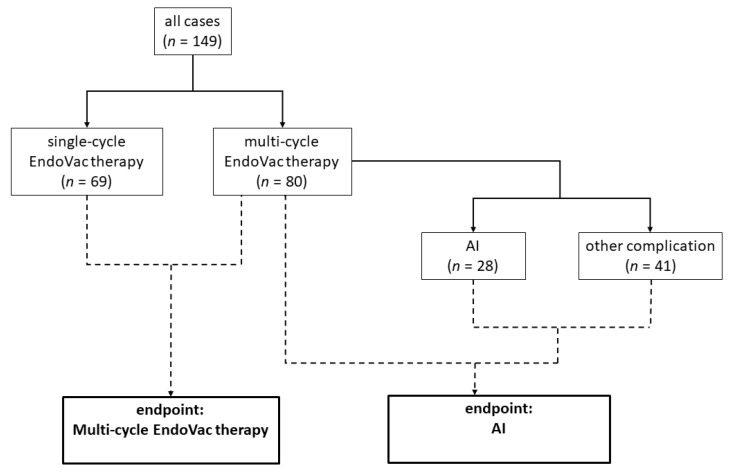
Overview of the data used in this study and the two endpoints. AI = anastomotic insufficiency.

Between September 2017 and March 2022, 149 patients underwent minimally invasive surgery for resectable esophageal cancer, followed by prophylactic EndoVac treatment. Of these, 124 patients were treated via RAMIE and 25 were treated via hybrid surgery. Cases were divided based on two endpoints: endpoint 1, division based on the event of an AI (cases with AI: *n* = 28, 18.8%), and endpoint 2, division based on the number of postoperative EndoVac cycles required (cases with multi-cycle EndoVac therapy: *n* = 80, 53.7%). An overview of the data and endpoints can be found in Figure 1. Common clinical cutoff values were chosen for the quantitative variables to realize group division.

We found the correlation between BMI category (<25 kg/m^2^ (normal), 25–30 kg/m^2^ (overweight), >30 kg/m^2^ (obesity)) and AI to be significant (*p* = 0.049). The correlation between Charlson comorbidity index category (<3 (moderate), ≥3 (severe)) and AI was also significant (*p* = 0.036). Additionally, we found the correlation between BMI category and multi-cycle EndoVac therapy to be significant (*p* = 0.004). Moreover, the relationship between blood pressure medication and multi-cycle EndoVac therapy was also significant (*p* = 0.038). Other patient characteristics were not statistically significant (see Table 1).

### 3.2. Perioperative Characteristics

Perioperative characteristics comprised surgery type and duration, intraoperative blood loss, and R status. The correlation between surgery duration (<360 min (short), ≥360 min (long)) and multi-cycle EndoVac therapy was significant, with *p* = 0.009 for the complete procedure and *p* = 0.004 for the thoracic part of the procedure (here <240 min (short), ≥240 min (long)). Other correlations were not statistically significant. Perioperative parameters are presented in Table 1.

**Figure 2:**
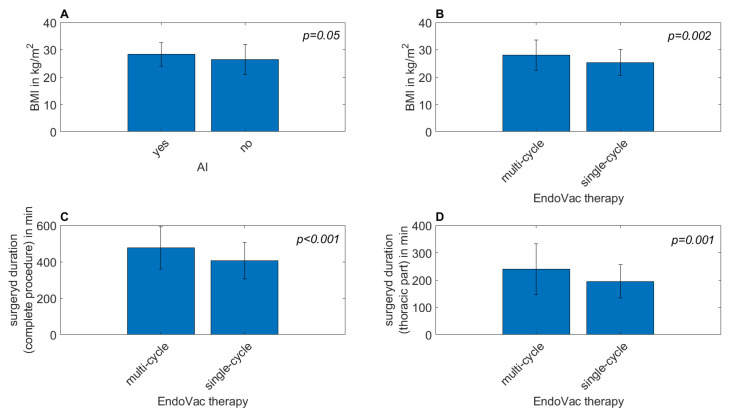
Bar plots of continuous variables with mean values of the different endpoints. The error bars denote standard deviations. (**A**) BMI for the AI endpoint, (**B**) BMI for the multi-cycle EndoVac therapy endpoint, (**C**) surgery duration of the complete procedure for the multi-cycle EndoVac therapy endpoint, (**D**) surgery duration of the thoracic part for the multi-cycle EndoVac therapy endpoint. Pairwise *p*-values comparing the distributions of variables for different event groups were computed using Welch’s *t*-test.

We also computed Welch’s *t*-test for continuous variables to test the averages of the two groups for equality for each endpoint. Details can be found in Table 2. We found a significant difference in BMI for cases with and without AI (*p* = 0.05) and cases with single- and multi-cycle EndoVac therapy (*p* = 0.002). We also found a significant difference between single- and multi-cycle EndoVac therapy for the complete (*p* < 0.001) and thoracic (*p* = 0.001) surgery durations (Figure 2).

### 3.3. Modeling the Likelihood of an AI and a Multi-Cycle EndoVac Therapy

We used the variables that were correlated with the endpoints to model the likelihood of the respective events. We performed a binomial logistic regression to determine the ability of BMI and the Charlson comorbidity index to predict the likelihood of an AI; the model was not significant (*p* = 0.107). Additionally, we performed a binomial logistic regression analysis to determine the ability of BMI, blood pressure medication, and surgery duration to predict the likelihood of multi-cycle EndoVac therapy. We performed a similar analysis using the duration of surgery on the thoracic part instead of the duration of the full surgery and found similar results. The binomial logistic regression model was statistically significant (*p* < 0.001). Balanced accuracy in classification was 66.7%, with a sensitivity of 72.5% and a specificity of 60.9%. Of the three variables input into the regression model, two, BMI (*p* = 0.048) and surgery duration (*p* = 0.004), contributed significantly to predicting multi-cycle EndoVac therapy, while the third variable, blood pressure medication, showed no significant effect (*p* = 0.090). For every unit increase in BMI, the odds of the patient being in the high-risk group were 1.074 times larger than the odds of the patient not being in the high-risk group when all other variables were held constant. For every unit increase in surgery duration, the odds of the patient being in the high-risk group were 1.005 times larger than the odds of the patient not being in the high-risk group when all other variables were held constant. Thus, both BMI and surgery duration had small but significant negative effects. For all model coefficients and odds, see Table 3.

### 3.4. Subgroup Analysis

We performed a subgroup analysis using only cases in which the variables above had values that defined the high-risk group. Significant results are presented in Table 4. Note that, unless otherwise specified, we used the cutoff values defined in Table 1. The subgroup analysis underlies the differences in BMI and surgery duration across differently defined high-risk and low-risk groups.

## 4. Discussion

Esophagectomy is a complex surgical procedure that involves removing and reconnecting parts of the esophagus to the stomach. There has been considerable technical progress in the field of esophagectomy in the last decade [6,15,17,18,36,37,38]. While minimally invasive techniques offer several advantages, including reduced postoperative pain and shorter hospital stays, there is still a risk of complications that can affect patient outcomes. Unlike other complications, the rate of AI has not substantially decreased with the transition from surgical approaches to minimally invasive surgery. It is expected that this will improve in the future through the use of modern technologies and artificial intelligence methods [39]. At present, AI is still a complication that needs to be addressed. One of the critical challenges in managing postoperative complications is the provision of adequate enteral nutrition to support the healing process and prevent complications such as AI, where the surgical connection between the esophagus and stomach does not heal properly. This study investigated risk factors for a complicated postoperative course and the subsequent need for PEJ tube supply for enteral nutrition. The high-volume center at Münster University Hospital implements pre-emptive EndoVac therapy after esophagectomy, which ensures uniform postoperative access and control. Therefore, this study provides a unique opportunity to examine risk factors using a limited but high-quality dataset.

The findings of this study reveal that BMI and the Charlson comorbidity index are significantly correlated with the occurrence of AI. These results are consistent with previous experiences of anastomotic healing [40,41,42,43]. Additionally, the surrogate parameter, multi-cycle EndoVac therapy, is correlated with BMI, blood pressure medication use, and the duration of surgery. High BMI and longer surgery durations were identified as potential risk factors for multi-cycle EndoVac therapy and a complicated postoperative course. A subgroup analysis hints at the importance of BMI and surgery duration, as well as ASA score, blood pressure medication, hemoglobin level, and neoadjuvant therapy type as indicators of a high-risk subgroup. BMI, surgery duration, ASA score, hemoglobin level, cardiovascular comorbidities, and neoadjuvant therapy are known risk factors for AI following gastrointestinal surgery [44,45,46]. Future studies of more extensive samples might confirm these features as risk factors for AI following esophagectomy.

The study’s results offer valuable insights into managing postoperative complications after minimally invasive esophagectomy. We found evidence that high BMI and prolonged surgery duration increase the risk of postoperative complications.

By identifying predictive factors, healthcare professionals can proactively address the nutritional needs of high-risk patients, potentially reducing the incidence of complications and improving overall postoperative outcomes. Pre-emptive placement of a PEJ tube can be justified in cases involving the factors we identified in this study. Despite these valuable findings, the study has limitations that need to be acknowledged. The study was conducted at a single center; therefore, the generalizability of the results may be limited, and multi-center studies are suggested for future research. The use of machine learning methods may allow the exploration of interactions between variables and the generation of more generalizable predictive models.

## 5. Conclusions

In conclusion, understanding and identifying risk factors associated with postoperative complications following minimally invasive esophagectomy can help improve patient care. The study’s findings emphasize the importance of personalized approaches in managing post-surgery complications and highlight potential areas for intervention to optimize patient outcomes. Ultimately, this research contributes to the ongoing efforts to enhance the safety and effectiveness of minimally invasive esophagectomy procedures.

## Figures and Tables

**Table 1:** Pre- and perioperative characteristics: Number of cases with the respective conditions with respect to the two endpoints investigated in this study. *p*-values correspond to a chi-square test and * denotes statistical significance. AI = anastomotic insufficiency, BMI = body mass index, ASA = American Society of Anesthesiologists, CRP = C-reactive protein.

	Endpoint: AI			Endpoint: Multi-Cycle EndoVac Therapy		
Preoperative characteristics						
	No AI	AI	*p*-value	Single-cycle	Multi-cycle	*p*-value
Demographic data						
Age in years			0.842			0.882
<65	67	14		36	45	
65–75	33	8		20	21	
>75	21	6		13	14	
Sex			0.118			0.657
male	94	26		54	66	
female	27	2		15	14	
BMI in kg/m^2^			0.092			0.004 *
<25	53	6		37	22	
25–30	41	13		21	33	
>30	27	9		11	25	
Preoperative diagnostics and therapy						
ASA score			0.744			0.094
<2	4	0		4	0	
≥2	117	28		65	80	
Tumor localization			0.423			0.625
upper or middle third	0	1		0	1	
gastroesophageal junction	121	27		79	69	
Neoadjuvant therapy			0.335			0.244
chemotherapy	47	6		29	24	
chemoradiotherapy	65	19		34	50	
none	9	3		6	6	
Charlson comorbidity index			0.036			0.709
<3	28	1		12	17	
≥3	93	27		57	63	
T-status pretherapy			0.234			0.281
T1	8	5		8	5	
T2	25	4		16	13	
T3	86	19		59	46	
T4	2	0		0	2	
N-status pretherapy			0.999			0.491
N0	27	6		13	20	
N+	94	22		56	60	
Medication						
Blood pressure medication			0.124			0.038 *
yes	69	21		35	55	
no	52	7		34	25	
Cortisone medication			0.999			0.542
yes	2	0		0	2	
no	119	28		69	78	
Immunosuppression			0.162			0.298
yes	1	2		0	3	
no	120	26		69	77	
Anticoagulant			0.395			0.224
yes	27	9		13	23	
no	94	19		56	57	
Laboratory parameters						
Preoperative CRP in mg/dl			0.999			0.999
<0.5	33	6		23	16	
≥0.5	23	4		16	11	
Preoperative leucocytes			0.473			0.679
<10,000	81	14		64	70	
≥10,000	33	9		4	7	
Preoperative hemoglobin in mg/dL			0.763			0.999
<12	37	10		22	25	
≥12	84	18		47	55	
Perioperative characteristics						
Treatment group			0.518			0.478
full-robotic	54	10		27	37	
hybrid-robotic	67	18		42	43	
Surgery duration (complete procedure) in minutes			0.430			0.009 *
<360	33	5		25	13	
≥360	88	23		44	67	
Surgery duration (thoracic part) in minutes			0.473			0.004 *
<240	81	14		52	43	
≥240	33	9		11	31	
Intraoperative blood loss in mL			0.597			0.081
<100	37	11		24	24	
≥100	49	10		27	32	
R status			0.999			0.999
R0	115	26		65	76	
R1	6	2		4	4	

**Table 2:** Details of continuous variables and results of Welch’s *t*-test based on the AI and multi-cycle EndoVac therapy endpoints. * denotes statistical significance. AI = anastomotic insufficiency, BMI = body mass index, CRP = C-reactive protein.

	No AI		AI		
	mean	SD	mean	SD	*p*-value
Age in years	64.23	9.89	66.19	9.29	0.33
BMI in kg/m^2^	26.44	5.52	28.36	4.30	0.05 *
Preoperative CRP in mg/dL	0.85	1.86	0.68	1.09	0.71
Preoperative leucocytes	6640	2120	6400	1460	0.50
Preoperative hemoglobin in mg/dL	12.56	1.45	12.77	2.20	0.64
Surgery duration (complete procedure) in minutes	436.80	107.37	473.07	137.17	0.20
Surgery duration (thoracic part) in minutes	214.69	80.92	240.91	91.59	0.22
Intraoperative blood loss in mL	246.51	358.69	304.76	470.03	0.60
	Single-cycle		Multi-cycle		
	mean	SD	mean	SD	*p*-value
Age in years	65.17	9.32	64.10	10.19	0.508
BMI in kg/m^2^	25.34	4.80	28.07	5.50	0.002 *
Preoperative CRP in mg/dL	0.90	2.14	0.70	1.01	0.621
Preoperative leucocytes	6350	1960	6810	2060	0.174
Preoperative hemoglobin in mg/dL	12.49	1.49	12.70	1.72	0.417
Surgery duration (complete procedure) in minutes	405.90	100.92	476.15	115.43	<0.001 *
Surgery duration (thoracic part) in minutes	195.13	61.11	239.50	93.74	0.001 *
Intraoperative blood loss in mL	230.39	373.78	283.04	391.02	0.482

**Table 3:** Model coefficients of the logistic regression used to determine the ability of BMI, blood pressure medication, and surgery duration to predict the likelihood of multi-cycle EndoVac therapy. * denotes statistical significance. CI = confidence interval, BMI = body mass index.

	Coefficient	Standard Error	*p*-Value	Odds Ratio	Lower 95% CI	Upper 95% CI
Intercept	−4.381	1.143	<0.001 *	0.013	0.001	0.118
BMI in kg/m^2^	0.072	0.036	0.048 *	1.074	1.001	1.15
Blood pressure medication	0.617	0.364	0.090	1.854	0.909	3.783
Surgery duration (complete procedure) in minutes	0.005	0.002	0.004 *	1.005	1.002	1.009

**Table 4:** Subgroup analysis investigating variables in the multi-cycle EndoVac therapy endpoint. Subgroups are defined by cutoffs resulting in subgroup sizes given in the left column. Only significant results are shown. AI = anastomotic insufficiency, BMI = body mass index, ASA = American Society of Anesthesiologists.

Subgroup Definition	Variable	*p*-Value
Endpoint: Multi-cycle EndoVac therapy		
BMI ≥ 25 (*n* = 90)	ASA score	0.018
	Blood pressure medication	0.038
Blood pressure medication (*n* = 90)	BMI category	0.003
	Hemoglobin preoperative	0.047
	Surgery duration (thoracic part)	0.005
Surgery duration (thoracic part) ≥ 240 min (*n* = 41)	Neoadjuvant surgery radiochemotherapy vs. chemotherapy or none	0.035
Surgery duration (full surgery) ≥ 360 min (*n* = 110)	BMI > 25	0.014
	ASA score	0.041
Charlson comorbidity index ≥ 3 (*n* = 120)	BMI category	0.021
Endpoint: AI		
Surgery duration (thoracic part) ≥ 240 min (*n* = 41)	Neoadjuvant surgery radiochemotherapy vs. chemotherapy or none	0.024

## Data Availability

All data are available in the main text.
